# The robotic-surgery propositional bank

**DOI:** 10.1007/s10579-023-09668-x

**Published:** 2023-06-13

**Authors:** Marco Bombieri, Marco Rospocher, Simone Paolo Ponzetto, Paolo Fiorini

**Affiliations:** 1 Department of Computer Science, University of Verona, Verona, Italy; 2 Department of Foreign Languages and Literatures, University of Verona, Verona, Italy; 3 DWS Group, University of Mannheim, Mannheim, Germany

**Keywords:** Medical natural language processing, Semantics, Semantic role labeling, Annotated corpus, Surgical data science

## Abstract

Robot-assisted minimally invasive surgery is the gold standard for the surgical treatment of many pathological conditions since it guarantees to the patient shorter hospital stay and quicker recovery. Several manuals and academic papers describe how to perform these interventions and thus contain important domain-specific knowledge. This information, if automatically extracted and processed, can be used to extract or summarize surgical practices or develop decision making systems that can help the surgeon or nurses to optimize the patient’s management before, during, and after the surgery by providing theoretical-based suggestions. However, general English natural language understanding algorithms have lower efficacy and coverage issues when applied to domain others than those they are typically trained on, and a domain specific textual annotated corpus is missing. To overcome this problem, we annotated the first robotic-surgery procedural corpus, with PropBank-style semantic labels. Starting from the original PropBank framebank, we enriched it by adding new lemmas, frames and semantic arguments required to cover missing information in general English but needed in procedural surgical language, releasing the Robotic-Surgery Procedural Framebank (RSPF). We then collected from robotic-surgery textbooks as-is sentences for a total of 32,448 tokens, and we annotated them with RSPF labels. We so obtained and publicly released the first annotated corpus of the robotic-surgical domain that can be used to foster further research on language understanding and procedural entities and relations extraction from clinical and surgical scientific literature.

## Introduction

Surgery is a medical specialty that uses operative manual and instrumental techniques on a person to investigate or treat a pathological condition such as a disease or injury, to improve bodily function or appearance, or to repair unwanted ruptured areas. The act of performing surgery is referred to as *surgical procedure*. In particular, robot-assisted minimally invasive surgery is the gold standard for the surgical treatment of many pathological conditions since it guarantees to the patient shorter hospital stay and quicker recovery (Taylor et al., 2016). The robotic-surgical scientific literature is constantly evolving, and every year several up-to-date, high-quality academic papers and textbooks are published. They are an essential study material for medical students and professionals. Each (robotic) surgical procedure well documented in the literature, contains information on which are the main actions, which instruments must be used, which anatomical parts are involved, which are the precautions that must be taken for an adequate treatment of the patient and to what surgical step they are related to. The anatomical patients differences and the changes to the general procedure to manage them, are also often described. A surgical procedure then contains temporal, spatial and causal information between surgical actions. In addition to intra-operative treatment, pre-operative precautions and post-operative care are also often described.

These texts are meant and written for the understanding of human readers and therefore present the information in unstructured natural language. Having algorithms capable of both understanding the surgical natural language and organizing the content in a more structured and processable form, would pave the way for developing intelligent surgical and clinical systems: extracting knowledge from text, it would be possible to develop decision-making or question-answering systems that inform autonomous surgical robots, or help the surgeon, assistant or trainee to remember the operating steps, their order, the instruments to be prepared before the procedure, the anatomical parts to operate and precautions to follow before, during and after the surgery. These intelligent systems could help the surgeon to optimize the treatment of the patients on the basis of their anatomical features and their pathologies by providing theoretical-based suggestions (Wang et al., 2013). Furthermore, specific surgical language understanding and semantic role labeling systems can also be used to improve the automatic generation of operative images’ captions (Zhang et al., 2021), a promising and active research-line also outside the biomedical domain (Bhattacharyya et al., 2022).

As much of this information comes in an unstructured form, Natural Language Processing (NLP) is crucial for transforming relevant information hidden in free-text into structured knowledge. As a large and complex domain, surgery uses a very specialized language, and the same concept may be expressed using semantically similar but syntactically and linguistically different expressions depending on the type of document or the writer’s background. Creating NLP models capable of understanding the complexity of surgical language is extremely useful in improving healthcare and advancing medicine, but having high quality and specialized available data is an essential requirement to advance in this line of research (Locke et al., 2021).

Although efforts have been made to adapt NLP models to medical language (Chen et al., 2018; Houssein et al., 2021; Locke et al., 2021), few have addressed the surgical subdomain, probably due to the lack of annotated data and the high costs involved in producing them. To fill this gap, with this paper we publicly release the first annotated dataset for the Semantic Role Labeling (SRL) task (Palmer et al., 2005) applied to the robotic-surgical domain. It consists of sentences describing surgical procedures manually annotated with PropBank-style frames (c.f., actions) and their semantic roles (c.f., the agent performing the action, the anatomical part being operated during the action, etc.). Sentences are taken as-is from multiple sources, i.e., different books and academic papers, and written by various authors, so to capture possible variations of language.

This paper substantially extends and completes our previous work (Bombieri et al., 2022), where we presented a framebank for the robotic-surgical domain, i.e., a PropBank-style repository collecting frames describing actions and participants used in the robotic-assisted surgical domain. That is, while in Bombieri et al. (2022) we presented the catalogue of semantic labels to be used, in this paper we contribute a fully annotated dataset exploiting these labels. More in details, the extension and the novel contributions of this work with respect to (Bombieri et al., 2022) include:

- the full, manual annotation of a textual dataset consisting of 1,559 sentences with the semantic labels of the framebank presented in (Bombieri et al., 2022);
- the description of the annotation process, including the annotators’ training and the annotation guidelines;
- the public release of the annotated dataset.

We believe that this first manually annotated dataset for surgical natural language understanding is usable for extracting ad-hoc semantic surgical and clinical information from real academic textbooks and papers and that it can pave the way for applying NLP in the surgical domain, with great benefit for human operators and clinical research.

The paper is organized as follow: Sect. 2 deals with related works. Section 3.1 presents the Robotic-Surgery Procedural Framebank (RSPF). Section 3.2 describes the method adopted to annotate sentences using RSPF labels. Section 4 reports statistics and details about the annotated dataset. Finally, Sect. 5 summarises obtained resource and describes future works directions.

## Background

Researchers traditionally have built NLP lexical resources targeting general-domain English, which is syntactically and semantically different from domain-specific usage (Wang et al., 2013) as well as other languages (Moeller et al., 2020). Therefore, these resources cannot be directly exploited in very specific domains or with other languages, and different methods have been proposed to adapt them to specific needs. Updating the semantic frames bank can be viewed as a data-driven way for adapting algorithms for general purpose domains to more restricted and specialized texts. This section summarises some works that have adapted the general linguistic resources to the specific domain or to languages other than English.

Many works on updating English frame banks have been carried out in various fields, such as the clinical (Albright et al., 2013; Wang et al., 2013), the biomedical (Chou et al., 2006; Majewska et al., 2021), and other non-biomedical domains such as software analysis and cooking recipes (Jiang et al., 2020; Wang, 2015).

Wang et al. (2013) has considered texts written in different laparoscopic cholecystectomy operational notes stating that the language is significantly different from general English and existing semantic resources have limited coverage of the action verbs that frequently occur in operative notes. Based on these observations, the authors have surveyed the usage of each verb in the sample dataset to determine the verb meanings and semantic arguments of each one. In this way, they have extracted a set of differently used verbs, and, following the PropBank guidelines, they have defined specific frames for them. This work, however, has considered only surgical, non-robotic procedures taken only from gastrointestinal surgery notes that use more schematic language than descriptions taken from textbooks used in our work. Finally, no annotated dataset with these newly defined frames was provided, hindering the possibility to benchmark available SRL tools on the considered domain.

Albright et al. (2013) has annotated clinical narratives with layers of syntactic and semantic labels to facilitate advances in clinical NLU. Following PropBank guidelines, new frames have been defined in a similar manner to the method described in our work. Although the dataset has dealt with a clinical language, our work considers a more specialised level, i.e., descriptions of robotic-surgical procedures, a restricted subset of the clinical domain considered by the paper (which includes for example disorders, physiology, chemicals and groups, anatomical notions, etc.). Unfortunately, the related dataset is no longer freely accessible due to copyright issues (Peng et al., 2020).

Chou et al. (2006) has presented a corpus of PropBank-style annotations for biomedical journal abstracts. The work has analyzed 30 biomedical verbs adding or modifying their meaning with respect to general English resources. Then, a semi-automatic method has been applied to annotate a collection of MEDLINE abstracts selected from the search results with the following keywords: human, blood cells, and transcription factors. First, predicate candidates were identified; then, an automatic tool was used to produce biomedical semantic roles; and, finally, the resulting annotations were manually corrected. In Majewska et al. (2021), a new resource that provides VerbNet-style (Schuler, 2006) frames for biomedical verbs is released, together with the presentation of key differences between the general and biomedical domain, and the design choices made to accurately capture the meaning and properties of verbs used in biomedical texts. The conclusion is that leveraging a specialized VerbNet helps systems to improve verb classification and thus to better tackle challenging NLP tasks in biomedicine. The two previous works have dealt with a biomedical language that is still very far from the procedural surgical one; moreover, the second one has dealt with VerbNet classes that are quite different from the PropBank frames adopted in our work.

Outside the medical domain, (Wang, 2015) has proposed a method for automatically extracting semantic information from software requirements specifications. First, frequent verbs were selected from software requirement specification documents in the e-commerce domain, to build the semantic frames for them. Then, selected sentences were annotated for using them as training material to benchmark different machine learning methods.

Jiang et al. (2020) instead has proposed a new annotated dataset for extracting information from recipes. The authors have defined ad-hoc entity types (action, food, tool, duration, temperature, condition clause, purpose clause and others) and relation types following the methodology of PropBank. Then a corpus was annotated and used to benchmark a neural span-based model extracting entities and relationship.

Finally, (Bakker et al., 2022) has applied a transformer-based SRL approach to map legislation from semi-free text to structured manually defined frames composed by fixed semantic roles. Obviously, the domain is completely different from the one of our paper, but the approach bears some similarity.

There are also several works proposing PropBank language-specific lexicons for languages other than English, both for specialized or general domain. For example, (Antony et al., 2020) has performed a SRL task in Tamil Biomedicine texts, extracting domain specific verbs and related semantic roles. Kara et al. (2020), Mirzaei and Moloodi (2016), Moeller et al. (2020) instead have built a general-domain PropBank specific for Turkish, Persian and Russian respectively. Jindal et al. (2022) has stated that despite the availability of SRL resources in different languages, it is almost impractical to build a single multilingual SRL labeler because of the differences in semantic labels and framebanks. To provide a possible solution to these issues, it has provided a family of auto-generated PropBanks for 23 languages from 8 language families, together with a small set of manually annotated sentences for Polish (100), Portuguese (3779) and English (16622), with the goal of enabling the construction of SRL models for resource-poor languages by annotating the text in different languages with a layer of universal semantic role labeling annotation.

**Fig. 1:**
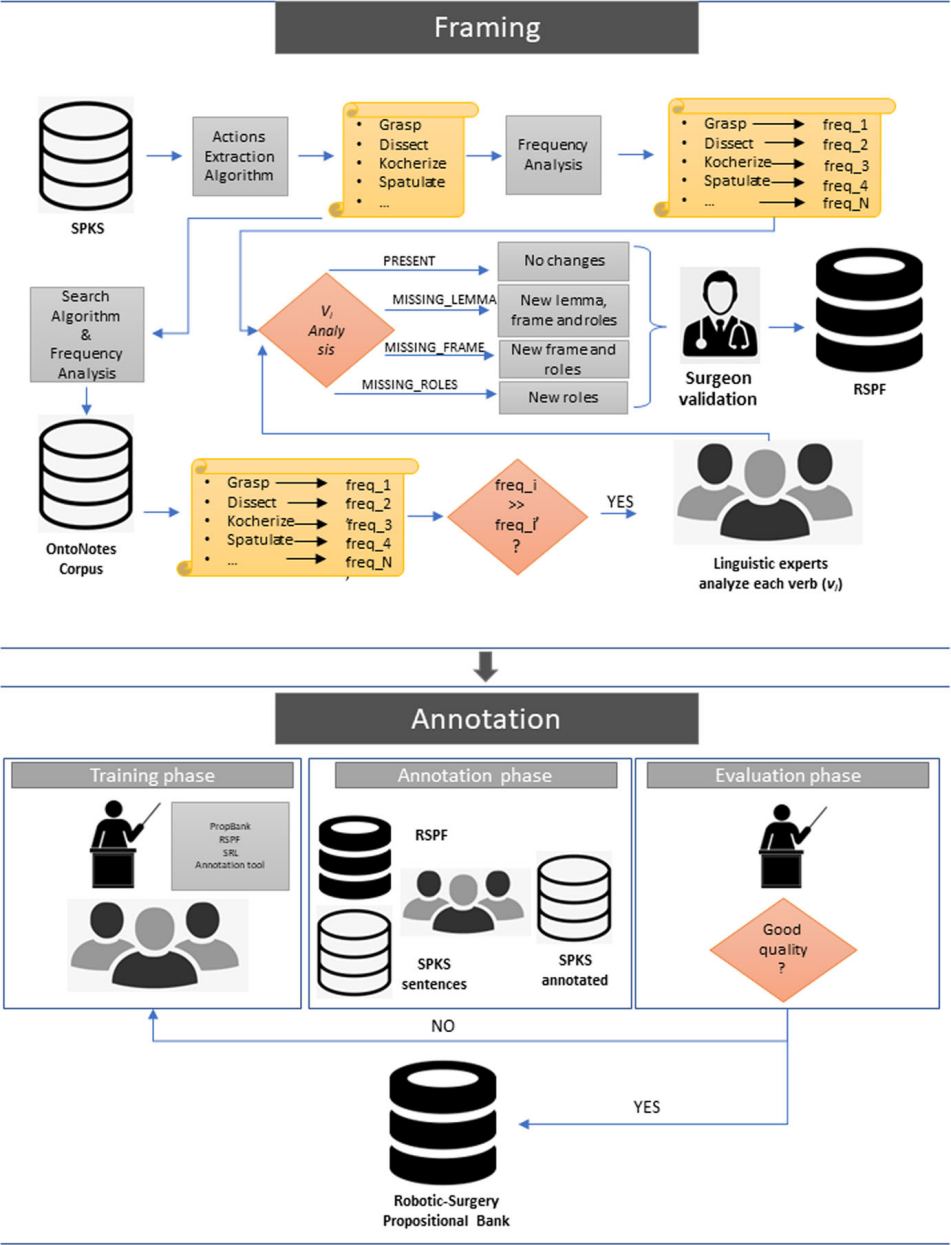
High level diagram of the method described in Sect. 3.1.4 for the framing of surgical domain verbs and annotation

## Building the robotic-surgery procedural propositional bank

The Robotic Surgery Propositional Bank (RSPB) is an extension of PropBank (Palmer et al., 2005) for the robotic-surgical domain. We recall that PropBank consists of:

- a *framebank*, i.e., a collection of *frames* (a.k.a., *meaning* or *senses*) for *lemmas* denoting predicates (*verbs* or *nominalized verbs*). Frames are specific to a given lemma, and each lemma has one  or more (*polysemous* lemma) associated frames. Moreover, each frame provides the specification of its semantic *roles*, i.e., the different labels that can be used to semantically characterize the arguments of the corresponding predicate.
- a corpus of text annotated (according to the framebank) with information about basic semantic propositions.

Following the steps described in (Palmer et al., 2005), also the development of RSPB is divided into two parts, namely the creation of a lexicon of frames files (RSPF, i.e., Robotic Surgery Procedural Framebank) summarised in Sect. 3.1), and the annotated dataset with RSPF’s labels, presented in 3.2.

Figure 1 shows a general overview of both steps: in the domain-verbs framing process, some automatic methods extract lemmas describing actions from robotic-assisted surgical texts. How often they appear in the target domain (*freq_i*) is compared to how often they appear in OntoNotes Weischedel et al. (2017) (*freq_i’*).  If *freq*_*i* $\gg$ *freq*_*i’*, i.e., the ratio between the two is greater than a fixed threshold, then the respective lemma is sent to a team of human linguistic experts that verify to which of the categories described in Sect. 3.1.1 the lemma belongs, modifying the corresponding frameset if necessary. The final frameset is validated by a clinician and publicly released. During the annotation step, a team of annotators is hired and trained. Annotation guidelines are written and sentences to annotate are provided. Then, the annotation process is performed. Quality checks are periodically carried out and, if necessary, the training step is resumed.

### The robotic surgery procedural framebank

For identifying procedural verbs and nouns used in the robotic-surgical domain, two strategies are applied. The first one (Sect. 3.1.3) deals with the detection of actions expressed by nominalized verbs, and it is based on keyword extraction. The second method (Sect. 3.1.4) is based on Part-Of-Speech (POS) tagging, and it is used to detect actions expressed by verbs. Their combination, together with additional low-frequency or missing candidates suggested by the clinician during the validation phase, offers a broad coverage of the robotic-surgery actions for considered domains.

#### Adapting PropBank to the robotic-surgical domain

RSPF is an adaptation of the latest release (version 3.1) of PropBank Palmer et al. (2005) to the robotic-surgical domain  This domain is substantially different than the general English one considered in PropBank, and thus this adaptation is necessary for extracting meaningful robotic-surgical procedural knowledge. By analyzing the semantic use of each lemma describing an action identified in the corpus (see Sect. 3.1.2 for details on the considered corpus) with respect to the PropBank framebank, each candidate is assigned to one of the four categories described in Table 1.

**Table 1:** Categories to which each of the candidate lemmas is assigned to

Category	Description
	The lemma is already present in PropBank and there is a frame file that adequately describes the use of the predicate. For this lemma, PropBank already describes appropriate semantic roles as core entities
	The lemma is already present in PropBank, and there is a frame file that adequately describes the use of the predicate. This frame, however, does not include domain-specific semantic roles often used in our domain
	The lemma is already present in PropBank, but a proper frame is missing, as the existing ones describe different meanings
	The lemma is not present in PropBank

If a lemma is assigned to the  class, no changes are needed, since PropBank already covers the robotic-surgical usage (i.e., there is a frame for the lemma that perfectly describes that usage of the predicate).

If a lemma is assigned to the  class, some semantic roles important for the robotic-surgery domain are missing, and therefore they must be added. The lemma *“to retract”* is an example of action belonging to this category. For it, PropBank offers the *“retract.01: to take back”* frame which actually covers the typical meaning of the surgical domain. However, only two roles are proposed for it:

- **Arg0:** *taker back, agent*
- **Arg1:** *thing retracted*

The verb *to retract* is however used very often in the robotic-surgery domain, together with additional information that allow to better describe the action: the instrument used for the retraction, the technique and/or manner, and the ending point or the indication of how much to retract. 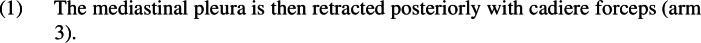


A candidate lemma may be assigned to the  class for two different reasons: i) the usage of the lemma is semantically and entirely different from all the frames covered in PropBank, and there is no overlap between the existing and new semantic roles; ii) the meaning is not completely new, but the existing frames are too broad to be useful for the robotic-surgery domain, i.e., the new frame deals with a subset of the meaning captured by (some of) the old ones. An example of the first case is the verb *“to grasp”*. For it, PropBank offers a single meaning *“grasp.01: to take hold of, comprehend”* with two semantic roles:

- **Arg0:** *grasper*
- **Arg1:** *thing grasped*

The robotic-surgical domain uses this lemma with a significantly different meaning, i.e., *“to clasp or embrace especially with the fingers or arms”*. For it, important information is also the grasper, the thing grasped, the instrument used for grasping, and important spatial indications for correct grasping. 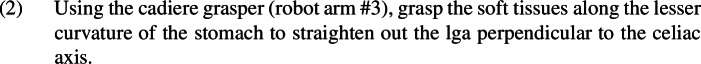


An example of the second case is the verb *“to approximate”*. For it, PropBank has the frame *“approximate.01: to be close or similar, cause to come near to or approach again”* with only two roles:

- **Arg0:** *entity coming close*
- **Arg1:** *entity coming close to*

It offers a broader meaning than the specialized one used in the robotic-surgery domain (*“to come near in position, to bring near”*), which is typically enriched with the following information: agent, entity coming close, entity coming close to, instrument and spatial indications.   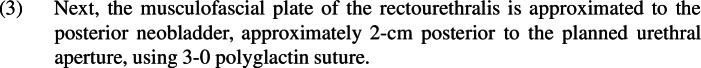

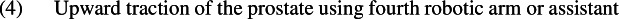


#### Collecting domain-specific lemmas

To extend PropBank to the procedural robotic-surgical domain, those verbs (or nominalized verbs) that are typical of the surgical domain have to be identified. The Surgical Procedural Knowledge Sentences (SPKS) dataset, presented in (Bombieri et al., 2021) is used to extract the domain actions of the procedural robotic-surgical domain. To the best of our knowledge, it is the only publicly available corpus of this domain.^1^ It describes 20 different robotic-surgical procedures belonging to four different disciplines: urology, gastrointestinal procedures, thoracic procedures and gynecology. For the comparison with general English we have instead considered the OntoNotes dataset. It is a large annotated dataset comprising various genres of text such as news, conversational telephone speech, weblogs, usenet newsgroups, broadcast and talk shows.

**Table 2:** (Left) Example of nominalized actions extracted using the method described in Section 3.1.3 with indication of the verb they refer to (“—” means missing corresponding verb) and the modification required. (Right) Example domain lemmas extracted using the method described in Sect. 3.1.4 with indication of the type of modification required

Nominalized actions	Verbs
<Placement , Place , >;	<Extraperitonealize , ${>}$
<Reflection , Reflect , >;	<Resect , ${>}$
<Retraction , Retract , >;	<Spatulate , ${>}$
<Exposure , Expose , >;	<Skeletonize , ${>}$
<Resection , Resect , >;	<Kocherize , ${>}$
<Mobilization ,Mobilize , >;	<Insufflate , ${>}$
<Traction , — , >;	<Redock , ${>}$
<Administration , Administer , >;	<Detubularize , ${>}$
<Identification , Identify , >;	<Grasp , ${>}$
<Excision , Excise , >	<Incise , ${>}$

The two methods presented in 3.1.3 and 3.1.4 are used to extract domain-specific actions from SPKS. For each domain-specific predicate, it is then necessary to check to which of the categories described in Table 1 the lemma belongs and proceed with framing as described in 3.1.5. Table 2 shows 10 examples of actions expressed by nouns identified by the method described in 3.1.3, and 10 examples of verbs identified by the method described in 3.1.4. For each of them, the indication of the type of modification that has been requested on PropBank is reported. Finally, as frequency-based methods for extracting domain terminology may miss some very specific terms rarely used in text (thus ensuring high precision but low recall), the final list of extracted candidate verbs and nominalized verbs was double-checked also by the clinician in the validation phase, for suggestion of possible missing domain-relevant verbs (and some examples of usage), thus improving the overall coverage of the domain. These additional verbs were then formalized in RSPF following the same framing process described in Sect. 3.1.5.

#### Finding frame-evoking nouns

In medical English, actions can be frequently expressed using nouns rather than verbs. For example, the noun *“suturation”* can be used to express an executable action. Below are two semantically equivalent sentences, where in the first the concept is expressed using a verb, and in the second using a nominalized verb: 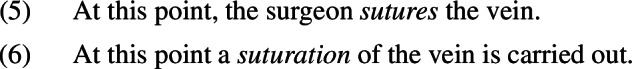


For nouns, the task of domain action detection is addressed as a keyword extraction problem, i.e., the identification of the lexical entities that best represent the domain according to a reference corpus. It is traditionally used in numerous fields to improve methods for browsing, indexing, topic detection, classification, contextual advertising and automatic summarizing of texts both with supervised and unsupervised approaches Hasan and Ng (2014). For our purposes, we have adopted the method proposed in Campos et al. (2020). It is an unsupervised approach (and thus it does not require annotated training data), built upon local text statistical features extracted from documents. It is corpus-, domain-, and language-independent, so it can be also adapted to the robotic-surgery field.

From the output of the algorithm, only nominalized verbs are selected. Since the most common morphological process involved in nominalization is derivation that can be defined as the creation of a new lexeme by the addition of an affix (i.e., a bound grammatical morphemes) (Varvara, 2017), obtained results are filtered keeping only those words ending with one of the following suffixes: “-sion”, “-son”, “-tion”, “ness”, “-ment”, “-ery”, “-ence”, “-ance”, “-ure”, “-ize”, “-ify”. False positives are finally removed from the list by manual revision.

#### Finding frame-evoking verbs

For verbs, a simple approach that compares the frequency of terms with an action role between the SPKS and OntoNotes corpora is used. For each token of the domain text, its POS tag (Bird, 2006) and the number of its occurrences are calculated. Finally, sentences of the corpus in which it is used are retrieved. Only the tokens whose POS tag denotes a verb (i.e., *VB*, *VBP*, *VBZ*, *VBD*, *VBN* or *VBG*) are retained.^2^ Lemmatization is then applied, and each token (e.g., *“cauterized”*, *“cauterizes”*, *“cauterizing”*) is associated to the corresponding lemma (resp., *“cauterize”*), aggregating number of occurrences appropriately.  For instance, this method identifies as “in domain” verbs like *“cauterize”*, *“detubolarize”* and *“extraperitonealize”*, because they are frequent in surgery and very rarely used in general English and therefore the ratio between the frequencies of these verbs in the two different domains is very high. On the other hand, the method recognizes verbs such as *“need”*, *“aid”* and *“see”* as “general English” because they appear in the two corpora with similar frequencies.

#### Framing of domain-actions

The processes described in Sects. 3.1.3 and 3.1.4 allow to obtain a list of domain verbs and nominalized verbs associated to a list of SPKS sentences where they are used. Each lemma and the respective sentences are then analysed by domain experts to understand to which of the categories described in Sect. 3.1.1 the lemma belongs to.

**Fig. 2:**
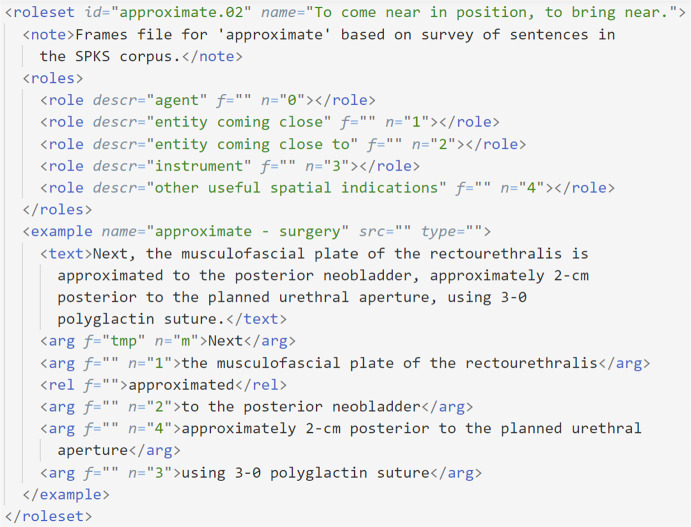
XML file for the “approximate” lemma. It contains the number of the frame (*02*), with its informal definition (*to come near in position, to bring near*). It then enumerates a list of semantic core roles (numbered from 0 to 4) and provides an annotation example

The framing was performed by three linguistic experts with a 3-year experience in the robotic-surgical domain and validated by a clinician. All frames are collected in XML files. Figure 2 is an example of the corresponding XML file for the lemma *approximate*.

In the case , the lemma is not present in PropBank, and thus it is an unknown word for the resource. Domain experts therefore perform the following actions: (i) they add the new lemma to the resource; (ii) they add a new frame to the inserted lemma; (iii) they provide a textual definition of the meaning of that lemma in the surgical domain taken from online medical dictionaries, in particular Webster Dictionary^3^ and The Free Medical Dictionary^4^; (iv) they add appropriate semantic core roles; (v) they add at least one example of SRL-style annotation for the new frame.

In the case , the lemma is already in the resource, but with inappropriate frames. In this case, domain experts perform only steps (ii)-(v).

In the case , the lemma is already in the resource with an appropriate frame, but with an inappropriate set of core roles. In this case, steps (iv-v) are performed.

Finally, in the case , the lemma is already in the resource, with an appropriate frame and core roles. None of the previous steps are performed.

During step (iv), a role is considered as core, if arguments playing that role occur with high frequency in the corpus’ sentences that use that lemma (i.e., it is present in more than 50% of sentences where the lemma is used)^5^ or, independently of its usage in the corpus, if it is considered fundamental by domain experts for the interpretation and representation of the action.

#### Framing effort

The framing step is quite expensive because it is carried out manually by personnel who must have expertise both in linguistics (SRL annotations in PropBank style) and in the robotic-surgical domain. The framing step took about 80 h to be completed.

### The robotic surgery procedural propositional bank

This section presents the annotation process of sentences from the robotic-surgical domain according to the frames and roles defined in RSPF. In particular, Sect. 3.2.1 introduces the annotation team. Section 3.2.2 discusses the texts to be annotated and their typology. Section 3.2.3 describes the annotators training process. Section 3.2.4 details the tool used to annotate the sentences and the adopted post-editing technique. Section 3.2.5 describes the annotation process and provides annotation guidelines. Finally, Sects. 3.2.6 and 3.2.7 discuss inter-annotation agreement and training effort respectively. Through these steps, RSPB has been created. RSPB wants to stimulate research in the field of natural language processing applied to robotic-surgical texts. In particular, it is designed for the SRL task, which is the basis of many NLP applications. SRL is traditionally framed as either a dependency-based (Surdeanu et al., 2008) or a span-based (Carreras & Màrquez, 2005) labeling task. Given a predicate in a sentence, the difference between the two settings is in the formalism used to represent its arguments. Span-based SRL requires the identification and classification of the entire textual span of an argument, whereas dependency-based SRL is concerned about labeling only the head of the argument. In the dataset developed in this work, sentences are annotated in a span-based fashion.

#### The team

A team of four people with different roles has carried out the annotation process. In more detail, the team is composed by:

- Two annotators. They are bachelor’s students of linguistics. During their studies they have already encountered issues related to semantic annotation of corpora and successfully passed the relevant exams. However, they never delved into PropBank style annotation. They have an excellent knowledge of the English language (C1 language level) but they have no knowledge of the medical domain. They were involved in the project with a student collaboration contract of 150 h each. They were exclusively concerned with the annotation work.
- The project leader. He is a PhD candidate in computer science. He deals with NLP issues applied to medicine. He has the same English language level of the annotators. He was in charge of training, coordinating and revising the annotation team by answering doubts and refining the guidelines based on annotation errors, and of setting up the annotation tool.
- The surgeon. He responded to the doubts collected and presented by the project leader.

The total of the sentences were annotated by the two annotators with the following proportions respecting the needs and timing of each: the first one annotated approximately $65\%$ of the sentences while the second the remaining $35\%$. During the annotation, the project leader revised approximately 1/5 of their annotations to find recurring errors and improve the guidelines accordingly. The annotators processed and labeled a different number of sentences at the same time: this shows that the task, due to the high concentration and the fatigue load lends itself to being carried out in a different way according to human characteristics and skills. Due to cost reduction strategy and financial possibilities, the surgeon was just involved in the role of answering doubts, instead of having him participating directly into the annotation process.

**Fig. 3:**
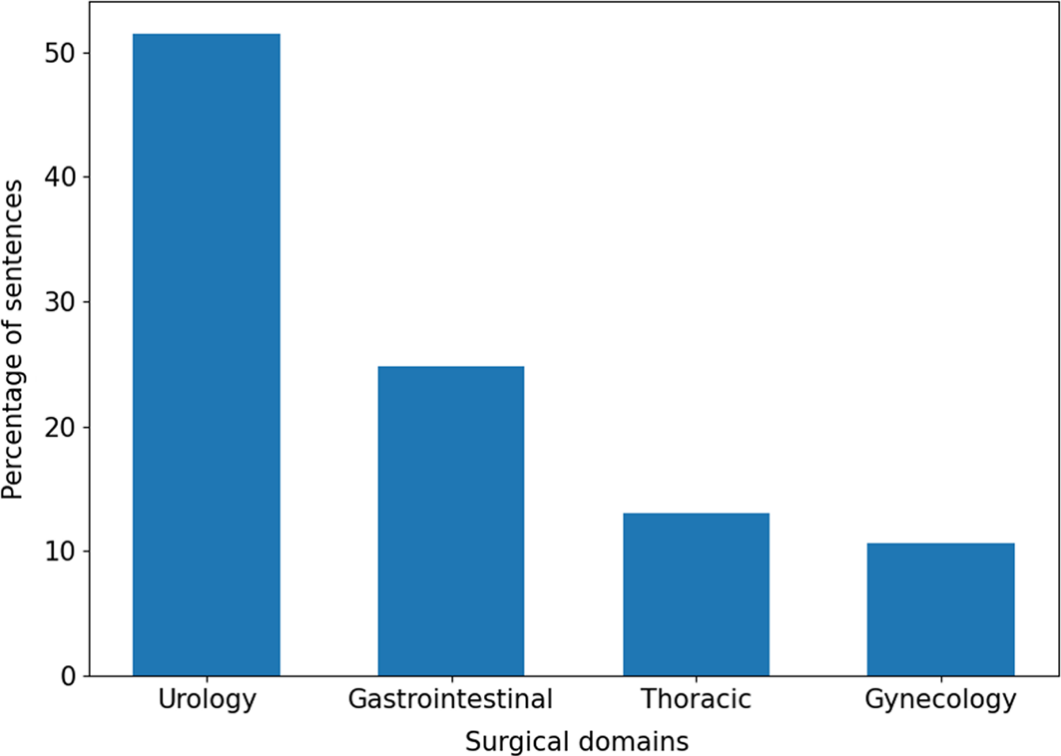
Surgical sub-domains annotated: *Urology* - 51.51% of the sentences; *Gastrointestinal procedures* - 24.82% of the sentences; *Thoracic procedures* - 13.02% of the sentences; *Gynecology* - 10.65% of the sentences

#### Text to annotate

The team annotated sentences of different surgical procedures, taken from different documents and written by different authors. They vary greatly in the writing style: the procedure descriptions are essential and schematic in some cases, while longer sentences enriched with background information are used in others. In more detail, we asked to annotate an extended version of the procedural part of SPKS dataset. In total, we relied on 1,559 annotated sentences describing 28 surgical procedures of four different robotic-surgery sub-domains, whose distribution is summarised in Fig. 3. All the sentences are therefore procedural, in the sense described in (Bombieri et al., 2021). Approximately $80\%$ of the sentences are taken from robotic-surgery textbooks describing how-tos of surgical procedures, while $20\%$ from academic papers or case reports dealing with academic research on surgical procedures or descriptions of real interventions on specific patients.

#### Training process

The annotators, despite having basic knowledge of linguistics and semantic roles annotation, did not know the PropBank style of annotating spans of text. In a first step, during two workshops of one hour each, the project leader introduced the annotators to the project, the ultimate purpose of these annotations, PropBank and PropBank style SRL annotation, and the annotation tool.

At the end of these workshops the annotators were asked to annotate 15 general English sentences of increasing complexity following the PropBank annotation guidelines. At the end, the annotation was evaluated by the project leader. The process was repeated with new sentences until a 90% inter-annotator agreement (IAA) with the project leader was reached, following a similar approach to the one presented in (Hovy et al., 2006).

Then, the project leader introduced RSPF to the annotators, focusing on the differences compared to PropBank. The same annotation experiments was conducted, but this time on surgical domain sentences instead of general English ones. Although the annotation guidelines are similar, this experiment was intended to measure the understanding of the surgical text by the annotators. The project leader analyzed and discussed the errors of the annotators and refined the guidelines providing them with more explanations to fill the doubts, until a $85\%$ inter-annotator agreement with the project leader was reached.^6^ Both arguments labeling and the choice of predicate’s meaning were evaluated.^7^

Then, during the actual annotation of the whole dataset, the project leader analyzed 20% of the sentences of the two annotators and organized weekly meetings with them discussing possible mistakes and answering their doubts. The annotators were then asked to revise the labeling if needed be, and to double-check the previous annotations in light of the new indications.

At the end of the dataset annotation process, 60 SPKS sentences were assigned to both annotators, which were asked to annotate them in parallel without confronting each other. The inter-annotator agreement on these annotations was calculated as reported in Sect. 3.2.6, both on predicates and arguments labels.

**Fig. 4:**
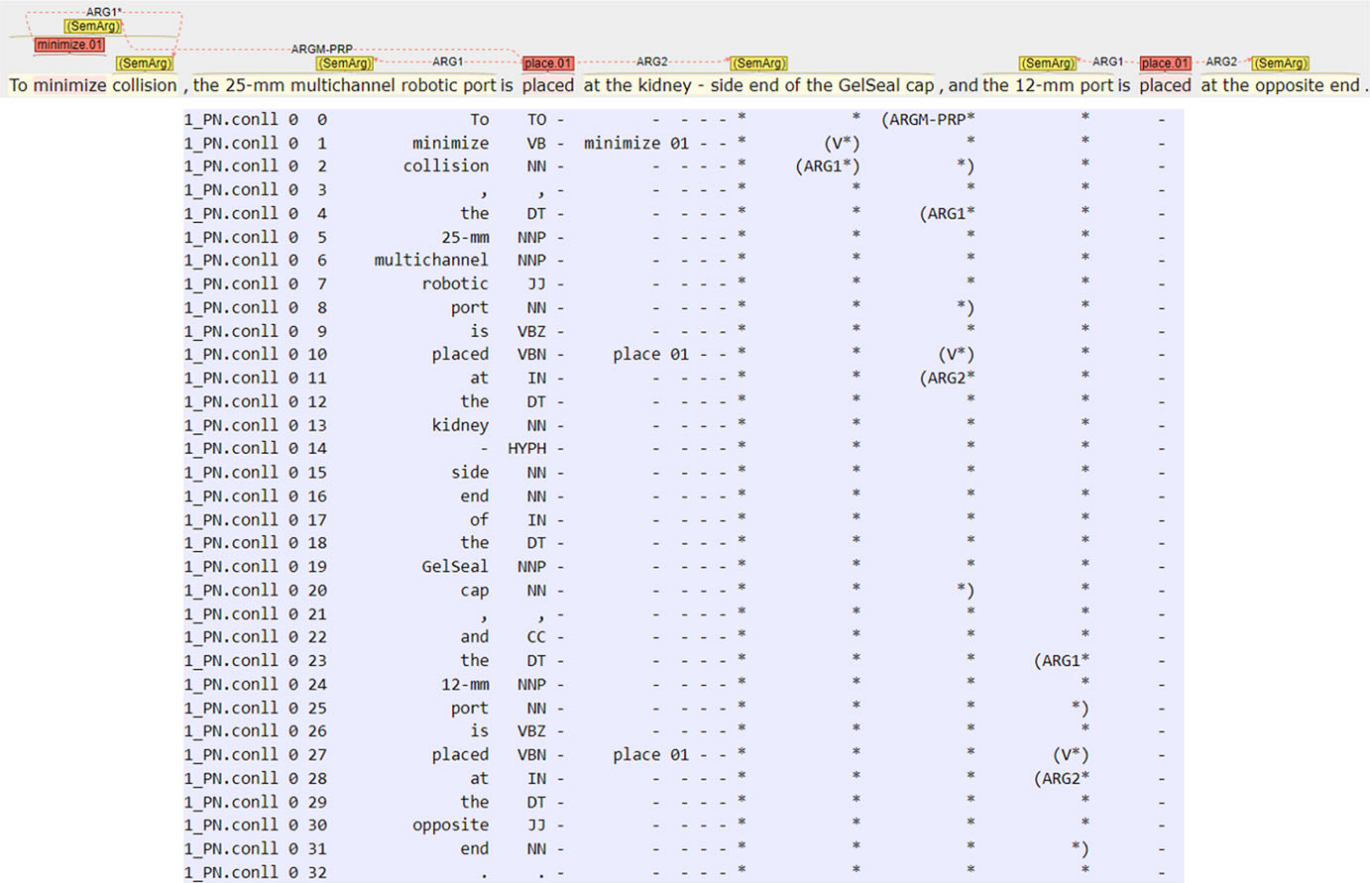
On top, annotation example of one sentence through Inception tool graphical interface. In red are predicate annotations while in yellow arguments related to the corresponding predicate. The annotation is finally exported in CoNLL-2012 format and it is directly processable by state-of-the-art SRL tools. Of the CoNLL-2012 fields, only the following columns have been annotated: the 3rd (identification number of the token), the 4th (list of tokens in the sentence), the 7th and 8th (predicate and corresponding frame number) and from the 12th to the second-last containing CoNLL-2012 annotations. In this sentence, three predicates are present: the first (minimize.01) is linked with only one argument (Arg1), the second (place.01) with two (ArgM-PRP, Arg1 and Arg2) and the third (place.01) with two (Arg1 and Arg2) whose meanings are contained in the RSPF. (Color figure online)

#### The annotation tool and post-editing technique

To reduce the annotation effort, a semi-automatic annotation approach was adopted. In a first step, the dataset was processed with a general English span-based SRL tool (Shi & Lin, 2019) for automatically obtaining PropBank annotations of the sentences in CoNLL-2012 format.

The annotations thus automatically obtained were uploaded on a server running Inception (Klie et al., 2018), a tool supporting SRL-style labeling of text. Inception has been setup to allow user friendly SRL annotation of the sentences. The annotators were asked to post-edit and revise the PropBank annotations according to RSPF and the given guidelines (see Sect. 3.2.5). That is, instead of having to manually annotate the sentences from scratch, the annotators were asked to revise (i.e., adding missing annotations, deleting wrong annotations, changing wrong PropBank frames and roles to appropriate RSPF ones) the automatically provided candidate annotations, so to substantially reduce the annotation workload.

Figure 4 shows an excerpt of the graphical user interface of the tool with an example of annotation, and the corresponding content in CoNLL-2012 format, which is directly readable by state-of-the-art SRL methods.

#### Annotation process and guidelines

The RSPB dataset follows the PropBank style of annotating predicates and semantic arguments (Palmer et al., 2005). Accordingly, similarly to PropBank, our corpus is a collection of sentences with verbs and nominalized verbs annotated with the corresponding sense id (also known as a “frameset” or “roleset” id in PropBank) in RSPF, together with their related semantic arguments.

These semantic arguments are labeled according to some predefined categories (e.g., Arg0, Arg1, Arg2) whose specific meaning typically varies according to the predicate considered. The set of roles of each predicate is outlined in the corresponding RSPF frame that gives both semantic and syntactic information about each sense together with correspondences between the number and semantics. Numbered arguments (e.g., Arg0, Arg1, Arg2) reflect either the arguments that are required for the valency of a predicate (e.g., agent, patient, benefactive), or those that occur with high-frequency in actual usage (e.g., instrument, surgical technique, important spatial constraints) as explained in Sect. 3.1. In addition to numbered roles, RSPF adopts also the same modifiers of PropBank (e.g., ArgM-TMP, ArgM-PRP). Differently from the numbered ones, modifier roles are general and not frame specific. Examples are ArgM-TMP, denoting temporal information, ArgM-PRP, related to the purpose of the corresponding predicate, and ArgM-ADV indicating general adverbial information. The annotation of sentences with this information creates a dataset, which can be used as training data for a variety of medical natural language processing applications. However, for the annotations to be reliable it is necessary to follow a rigorous annotation process and precise guidelines. Since our corpus is a specialization of PropBank to the surgical domain, it therefore inherits a good part of the annotation guidelines from it.

The main tasks of the Robotic-Surgery Propositional Bank annotation are:

(i) to identify the predicates of the sentence if not already labeled by the automatic tool.
(ii) to choose a sense in RSPF for each predicate or verify if the one automatically assigned is correct;
(iii) to label core arguments for each predicate or verify if the labels automatically assigned are correct.
(iv) to label modifiers arguments if present or verify if the labels automatically assigned to them are correct.

For each sentence, step (i) is related to the predicate-level annotation. The annotators have to check the correctness of the automatically identified predicates, as well as to identify missing annotations (i.e., predicates not tagged as such by the automatic tool). If the algorithm has marked as a predicate a token that does not cover this role, it must be removed together with all the annotations of the arguments related to it. This case is quite rare since state-of-the-art algorithms tend to have a rather high ability to identify predicates. Examples that can sometimes mislead algorithms are those that contain highly-specialized domain expressions such as *running suture* which in surgery indicates a particular technique for closing the deep portion of surgical defects under moderate tension, while an algorithm not trained in medical language . Furthermore, the tool we used for automatically generating the candidate annotations also annotated modals and copulas. The annotators were asked to remove them (as verb) since for the purpose of procedural knowledge extraction, i.e., the extraction of surgical *actions* and semantic arguments linked to them, we deemed them not useful. Annotations of the modals have been kept however at the modifier argument level (with arguments ArgM-MOD) because they can be helpful to specify the obligatory nature of the corresponding action. Finally, in this step also some nominalized verbs, i.e., those nouns that refers to actions, have been annotated as predicate. At this point the step (i) is finished and annotators continued with the step (ii).

Step (ii) is still related to the predicate-level. At this point the annotators have a list of predicates to disambiguate using the corresponding RSPF file. For most of the general English predicates, the automatic tool will have already proposed an appropriate sense which must only be verified by the annotators. If for it RSPF distinguishes two or more verb senses, annotators are asked to choose the one that best suits the context. In some cases the process is straightforward because RSPF has only one available sense. This is the case of  predicates, either specific of the surgical domain (e.g., *skeletonize*, *detubularize* or *kocherize*) or general English ones (e.g., *accomplish* or *avoid*). In other cases, the disambiguation is more complex because there are multiple senses in RSPF. Cases of this type can be further divided into two sub-categories:

- one of the lemma’s senses is specifically used just in the surgical domain, while the other general English senses are rarely used in surgical procedural texts. An example is the lemma *grasp*, for which RSPF has two senses: *grasp.01: “to take hold of, comprehend”* clearly related to a general English usage; *grasp.02: “to clasp or embrace especially with the fingers or arms”* specific to the surgical use. In this case the disambiguation is typically straightforward, as the general English sense is not or only rarely used;
- the lemma has both general English and surgical-specific senses that may both occur in surgical procedural texts. An example is the lemma *follow*, for which RSPF has 9 senses. Although sense 09 *“move behind in the same direction”* has been added for surgical purposes, other general English senses are used as well in surgical texts, for example the 01 *“be subsequent, temporally or spatially”*. The disambiguation in these cases is more complex and the annotators are asked to reflect well on the meaning of the sentence, comparing it with available examples in RSPF, and to discuss with the project leader if necessary.

During step (ii), occasionally, annotators may come across predicates that do not have yet an existing entry in RSPF.^8^ In these cases, annotators are instructed to contact the project leader describing the situation and reporting the corresponding dataset’s sentence. The project leader analyzes the corresponding sentence and lemma and then he decides whether to add this lemma to RSPF (because it is a lemma with a surgical sense that was not covered in the initial construction of RSPF), or to ignore the case (when the lemma is only a rarely used surgical slang). The project leader may also consult the surgeon to take an informed decision.

Once the correct meaning of a predicate has been identified, annotators proceeded with step (iii), the argument-level annotation. While Arg0 is typically relative to the one who performs the action and Arg1 typically relative to the one who undergoes it, for the other numbered arguments RSPF has to be checked more carefully. The annotators has to analyze all the arguments (both core and modifier) automatically identified by the tool, as well as possibly arguments in the text not annotated by the automatic pre-processing, which are then added from scratch by the annotators. For core arguments, if the annotation label is not correct, the most appropriate numbering has to be inserted. For the arguments automatically labeled as modifier, the annotators have to check if a more appropriate core role is available in the roleset of the frame, and, if so, to replace the modifier with it.

Since the tool used for obtaining the first draft of the annotations is trained on general English text, i.e., it does not know the RSPF specific frames and roles added in the extension of PropBank, the case of spans annotated automatically with a modifier (for PropBank) instead of core role (for RPSF) is quite frequent. 
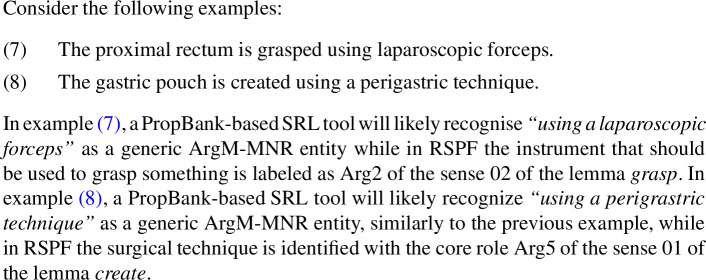
9

RSPF contains annotation examples also at this level to help annotators. In most cases, choosing a role is straightforward, given the verb specific definition of the label in the frame files. However, in some cases, it may be difficult to understand how to annotate a span of very specialized text. The annotators have to decide between the available labels based either on the explanations/examples provided in RSPF or by searching online the meaning of unknown domain words. If the doubt persists, the project leader is consulted.

During step (iv), for modifier arguments not to be translated into an RSPF core role according to step (iii), annotators are asked to verify whether the annotations proposed by the automatic tool is consistent with the guidelines of the original PropBank, and if not to correct them. RSPF does not add new modifier tags to PropBank, so no changes to its guidelines were necessary for these aspects.

Regarding which token to include in the span of the annotation (c.f., span boundaries) and corresponding exceptions, the same indications as in the PropBank’s guidelines are given to the annotators.

#### Inter-annotator agreement

Agreement between the two annotators has been measured at the end of the process on a sample of 60 sentences annotated by both, using the kappa statistic (McHugh, 2012), which is defined with respect to the probability of inter-annotator agreement and the agreement expected by chance. The kappa statistic has been computed both for predicates and arguments, obtaining the values 0.89 and 0.88 respectively. These values denote an almost perfect level of agreement between the annotators, reassuring on the adequacy of the annotation process and guidelines.

#### Annotation effort

The training and annotation process required a total of 450 h. Each annotator was employed for 150 h. In particular, the annotators were asked to annotate for a maximum of 1 h per session to reduce errors due to fatigue or boredom from the repetitive task. The project leader coordinated the annotation work for a total of another 150 h. In total, the whole process required 6 months to be carried out. Additional effort was required to setup the annotation tool and to write down the first version of the guidelines.

## The robotic-surgery PropBank

Both the framebank and the dataset resulting from the annotation process described in Sects. 3.1 and 3.2 are publicly available.^10^ This section presents and discusses some statistics about them. In more detail, Sect. 4.1 presents the RSPF, while 4.2 the annotated dataset.

**Table 3:** Semantic type of the core roles added to modified lemmas

Type	Description	Subtype	Number
Who and What	Core-role roles indicating who (or what) performs the action and who (or what) instead undergoes it. Often they respectively coincide with the robotic or the human operator and the anatomical part that is object of the action	Agent Patient	44 46
How	Core-role arguments indicating how the action is performed by specifying the surgical technique or the manner to follow to carry out the action, or the instrument to use	Manner or technique Instrument used	36 30
Spatial information	Core-role arguments specifying different kind of spatial information to know during the execution the corresponding action. These core-roles reply to questions “where?” or “through which passage or port?” or “starting from where?” or “ending where?” or final other frame-specific information such as orientation or spatial constraint to follow for safety reasons	Where Through Starting point Ending point Other	22 9 2 4 32
Purpose	Core-role argument explicitly describing the purpose of the main action. It is inserted as core-role only if very frequently present in our sample sentences	—	6
Other	Core-roles very specific to a particular lemma and thus not fitting in any of the above classes	—	13

### The framebank (RSPF)

Using the method described in Sect. 3.1, 252 lemmas have been analysed. At least one modification among those described in Sect. 3.1.1 have been requested in 109 cases. In particular, of the 252 analysed lemmas, 24 belong to  case, i.e., new lemmas (verbs or nouns) that describe very specific actions of the surgical domain not yet present in the original PropBank have been added. 22 lemmas belong to  case, i.e., new senses have been added to existing lemmas describing meanings not already covered by PropBank. Finally, 63 lemmas suffer of  problem, and thus corresponding existing predicate’s sense has been enriched with new semantic roles frequently used in robotic-surgery. Considering both the new lemmas added, new frames added to existing lemmas and the new core roles added to existing frames, a total of 244 core roles have been added. They are able to describe the surgical sense of the corresponding lemma in finer granularity. Table 3 shows the semantic type of core roles added for the robotic-surgical domain lemmas. The table considers all core roles added, both in existing frames and in new frames: while the number of core roles added in the first row of the table is quite high, most of them are actually due to  and , i.e., from frames not yet present in the orginal PropBank.

The nature of the semantic roles inserted highlights that, in the surgical procedural language, it is of utmost importance to indicate for each action that describes an operation, who or what performs the action (Arg0), the anatomical part that undergoes the action (often Arg1), the instrument with which to perform the action, the surgical technique to adopt, the purpose, and a series of spatial information that helps locate the target anatomy within the human body. Overall, the number of newly introduced and modified lemmas and frames indicates that the extension of PropBank to cover the robotic-surgical domain is substantial and that procedural surgical language differs from general English in terms of both predicates used and roles required.

**Fig. 5:**
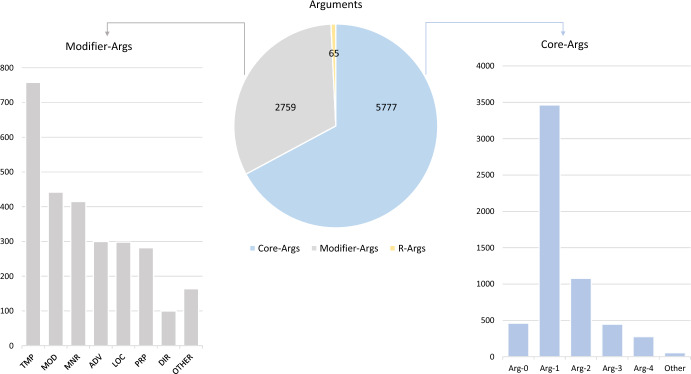
Arguments-level annotations. The pie-chart in the center shows the distribution of semantic arguments between modifier, core and references in our annotated dataset. In total, annotated 5,777 core arguments, 2,759 modifier arguments and 65 referent of other core arguments

### The annotated dataset

#### Dataset-level statistics

By following the annotation process and guidelines described in Sect. 3.2, the first annotated dataset specific for SRL of robotic-surgery textbooks was obtained. 28 different surgical operative descriptions have been annotated, for a total of 1,559 sentences and 32,448 tokens. The obtained dataset is composed of 12,202 annotations. Of them, 3,601 are predicate-level annotations, and 8,601 are argument-level annotations, both core and modifier. Figure 5 shows more detail about the distribution of core and modifier arguments of this dataset. An high percentage of the modifier arguments (left side of the figure) in the annotated dataset is covered by TMP. It provides temporal relationships between predicates and thus it is useful to give a chronological order to the actions that must be performed for a correct execution of the robotic-surgical procedure. There are also many tokens annotated with the MOD label: mostly tokens likes “can”, “must”, “might”, “may”, “would” are annotated in this way. Specifying these arguments is useful for extracting information on the mandatoriness of surgical actions, or events that may occur in certain circumstances. Finally, other frequent modifier arguments are MNR, which enrich the corresponding predicate with generic information about the manner with which an action should occur, and ADV which in our dataset mostly identifies span of texts containing conditional operators (if, then, else or otherwise). The identification of spans of text tagged with this label is important for the automatic reconstruction of a workflow from text, i.e., to represent the surgical process in a more structured and schematic way. The remaining arguments describe spatial, purposeful, or other information not labeled with any core role.

Among the core arguments present in the dataset (right side of the figure), the most frequent is Arg1. Unlike the other core arguments, it has a well-defined semantics. It plays the role of patient, i.e., the object that undergoes the action described by the predicate to which it belongs. Also Arg0 has a , (i.e., the agent who performs the action described in the corresponding predicate), but it is not so frequent in this dataset. This observation was also made in (Wang et al., 2013): in most cases, the agent did not occur in sample sentences as most actions in procedural language are described in a passive voice, and the agent in operative notes, or procedural textbooks, that is typically the surgeon, the assistant, or the robot, is omitted from the text. For the core arguments of higher number, there is not a well-defined semantics, since it varies according to the frame considered. However, Arg2, Arg3 and Arg4 are also frequent, and they are often associated with the surgical instrument, technique or spatial information.

The statistics reported in this section confirm that a procedural surgical description is typically composed of the specification for each action of an agent, a patient, a surgical instrument or technique, spatial information and other procedural attributes (e.g., temporal information and purposes).

#### Procedure-level statistics

As stated before, 28 different robotic-surgery descriptions have been annotated. The average number of sentences per procedure is approximately 56. The shorter description is 10 sentences long, while the longer is composed of 123 sentences. These values are very different from that of other *procedural* descriptions. For example, in (Mysore et al., 2019) a dataset of nano-material syntesis *procedural* descriptions is presented, and they reported 9 sentences per procedure on average. In (Zhang et al., 2012), *procedural* corpora about kitchen and automotive domains were presented, and an average of 12 sentences per description was reported. This means that the robotic-surgical procedures described in textbooks can be much longer and detailed than the procedural descriptions of other domains and sources, at least the ones considered so far in the literature.

Finally, our procedures have a mean of approximately 129 predicates (with a minimum of 21 and a maximum of 257) and are composed of a mean of $1,\!161$ tokens (with a minimum of 201 and a maximum of $2,\!457$).

#### Sentence-level statistics

A sentence of the robotic-surgery procedural domain has 2.31 predicates on average (minimum is 1 and maximum is 9) and is composed of 5.52 arguments on average (minimum is 1 and maximum is 20). Finally it has 20.81 tokens on average (minimum is 5 and maximum is 81). This last value can be compared with (Mysore et al., 2019), where the authors observed that a sentence for nano-material synthesis has 26 tokens on average, and with (Zhang et al., 2012), where the authors reported that a procedural sentence of the kitchen or automotive domain is composed of 12 tokens on average. Also this comparison suggests that depending on the domain, author, source, and purpose, there exist more or less complex *procedural* sentences, and those from robotic-surgery textbooks tend to be among the most complex ones.

**Table 4:** Examples of 10 different predicates with the indication of the number of senses with which they appear in the dataset (S. in text), the number of senses in the RSPF (S. in RSPF), and the reference to the most frequently used sense with the corresponding percentage of occurrence

Lemma	S. in text	S. in RSPF	Most frequent sense (% of occurrence)
Follow	4	9	[01] be subsequent, temporally or spatially (60.98)
Come	3	9	[01] motion (60.00)
Pass	3	11	[08] push through a passage (91.18)
Keep	3	6	[04] maintain some prepositional relationship (54.55)
Use	2	3	[01] to take advantage of, utilise (99.67)
Locate	2	2	[01] (cause to) be located in (66.67)
Introduce	2	3	[03] To put or place into something, to insert into (99.92)
Start	2	5	[01] Start (99.94)
Stop	1	3	[01] Stop, putting a stop to (100.00)
Enter	1	2	[01] Enter, go in (100.00)

#### Predicate-level statistics

In total, this dataset uses 452 different predicates labels. Of them, 410 are used with only one sense, while 42 can be used with different meanings. In more detail, $100\%$ of  lemmas are , meaning that there are not multiple surgical senses for very specialized, domain lemmas; furthermore, approximately $70\%$ of  lemmas and $85\%$ of  lemmas are used with only one sense in our dataset. These statistics show that the procedural surgical language makes extensive use of  predicates. Furthermore, even for lemmas with multiple senses available in RSPF, one of them is typically used much more frequently in the robotic-surgery domain than all the other senses. More in details, for each predicate *p* present in the dataset with at least two different meanings, denoting with $\alpha _{p}$ the frequency of the most common sense for the analysed predicate with respect to the total number of occurrences in the dataset, we observe that $\alpha _{p}$ is on average 0.77: that is, the most frequent sense is used in almost 8 times out of 10 of the occurrences of that predicate in the dataset, confirming that also for polysemous RSPF lemmas, only one sense is mainly used in the dataset.

Table 4 shows examples of predicates, with the specification of the number of senses with which they appear in the dataset, together with the information on the most frequently used sense and the corresponding percentage of occurrence.

Finally, from a tenses point of view, approximately $56\%$ of annotated predicates are in passive or past tense, $25\%$ are in a present or imperative tense, $14\%$ in present participle or gerund form and $5\%$ in a nominalized form. This states that the most common way to describe a surgical procedure is by using the passive, past, imperative or present tenses.

### Preliminary experiments with the obtained dataset

To confirm the need of adapting PropBank to better support information extraction and NLU within the robotic-surgery domain, a preliminary assessment was performed, to estimate how many of the dataset sentences would be wrongly or incompletely annotated by a state-of-the-art SRL tool, trained on PropBank and general English text, due to missing robotic-surgery specific information in the original PropBank:

- $\sim$36% of the sentences contains at least an action (with a corresponding frame in PropBank) and the mention of a domain-specific semantic role not in PropBank ( );
- $\sim$21% of the sentences contains at least an action describing a different meaning than the ones covered by its frames in PropBank ( );
- $\sim$2% of the sentences contains at least an action whose lemma is not in PropBank ( );
- the remaining sentences ( $\sim$41%) could be fully annotated with information already contained in PropBank ( ).

That is, more than half of the sentences would need some information covered by RSPF, but missing in PropBank, to be properly annotated.

To complement this investigation, we processed the contributed annotated dataset with a state-of-the-art, transformer-based, general-English SRL tool (Li et al., 2020), and compared the automatically provided annotations (the tool was trained only on the catalogue of frames and role in PropBank) with the manual ones (which comprise frames and roles also in RSPF). More in details, we measured the capability of the tool to correctly identify and disambiguate the semantic roles of the predicates, the standard measure used to assess SRL performance, obtaining an F1-score of 0.701. On general English text (CoNLL-05, CoNLL-12), the same tool achieves an F1-score above 0.87. This substantial difference confirms once again that the robotic-surgical language is noticeably different than the general English one, and that state-of-the-art SRL tools achieve a much lower performance score when applied on very specific domain, than the ones reported on standard general English benchmarks.



## Conclusions and future works

In this paper, we presented the first annotated resource for improving robotic-surgical natural language understanding. The dataset consists of a corpus collecting sentences from textbooks and academic papers, describing different robotic-surgical procedures, that have been manually annotated in a PropBank-style exploiting an extension of its framebank, i.e., the catalogue of frames and roles, for the robotic-surgical domain. In details, the construction of the dataset followed two steps: in the first one a framebank specific to the surgical domain (RSPF) has been defined. In the second step, RSPF was applied to manually annotate sentences, taken as-is without modification, from robotic-surgical texts. The annotation was performed at two levels: predicate-level, where predicates are identified and disambiguated with respect to RSPF, and arguments-level, where the same tasks are performed for the semantic arguments of each predicate. To perform the annotation, a team of collaborators with different roles has been setup: two annotators, one project-leader, and one clinician for a final validation. The annotators were duly trained on PropBank, SRL, and RSPF, both with theoretical workshops and with an iterative training process described in the paper. The resulting resource (RSPF and annotated dataset) is publicly released, to foster further research in this direction and to enable benchmarking of SRL tools in the robotic-surgery domain, yet little-explored for natural language understanding.

 Bombieri et al. (2023), we will leverage the annotated dataset to compare different machine learning methods. We aim to show how these annotations can be used, not merely to improve natural language understanding models for the surgical domain, but also for developing knowledge-based smart clinical applications. We will also work on further the resource with new annotations.

## Data Availability

The dataset presented in this paper is publicly released at https://gitlab.com/altairLab/robotic-surgery-propositional-bank. The web page contains a form to fill in to request the dataset. The form asks for name, surname, and institution and to adhere to the terms of the license (non-commercial academic research use).

## Footnotes

1 https://gitlab.com/altairLab/spks-dataset.

2 We only keep the verbs in this phase because the nouns describing actions have already been extracted with the method described in Sect. 3.1.3.

3 https://www.merriam-webster.com/medical.

4 https://medical-dictionary.thefreedictionary.com.

5 If for a lemma the associated sentences are less than 5, experts are instructed to retrieve additional examples through web search.

6 We targeted a lower threshold for the agreement to balance the high specificity of the surgical domain and the annotation costs.

7

8 In some cases, this situation may occur due to some lemmatization error of the automatic SRL tool providing the candidate annotations, something that can be easily fixed by the annotators by choosing the correct lemma and sense in RSPF.

9 These two examples show one of the benefits of RSPF over PropBank, for the surgical domain: it allows to better discriminate, with specific core roles, instruments and techniques, two substantially different entities in the surgical domain, which otherwise will be indistinguishably merged in the ArgM-MNR modifier role in PropBank.

10 https://gitlab.com/altairLab/robotic-surgery-propositional-bank.

## References

[CR1] Albright, D., Lanfranchi, A., Fredriksen, A., Warner, W. F. S., Hwang, J. D., Choi, J. D., Dligach, D., Nielsen, R. D., Martin, J. H., Ward, W. H., Palmer, M., & Savova, G. K. (2013). Towards comprehensive syntactic and semantic annotations of the clinical narrative. *Journal of the American Medical Informatics Association,**20*(5), 922–930. 10.1136/amiajnl-2012-001317 23355458 10.1136/amiajnl-2012-001317 PMC3756257

[CR2] Antony, J.B., Paul, N.R.R., & Mahalakshmi, G.S. (2020). Entity and verb semantic role labelling for tamil biomedicine. In B. R., P., Thenkanidiyoor, V., Prasath, R., Vanga, O. (eds.) Mining intelligence and knowledge exploration, pp. 72–83. Springer

[CR3] Bakker, R., van Drie, R.A.N., de Boer, M., van Doesburg, R., & van Engers, T. (2022). Semantic role labelling for dutch law texts. In Proceedings of the Language Resources and Evaluation Conference, pp. 448–457. European Language Resources Association. https://aclanthology.org/2022.lrec-1.47

[CR4] Bhattacharyya, A., Mauceri, C., Palmer, M., & Heckman, C. (2022). Aligning images and text with semantic role labels for fine-grained cross-modal understanding. In Proceedings of the Language Resources and Evaluation Conference, pp. 4944–4954. European Language Resources Association. https://aclanthology.org/2022.lrec-1.528.

[CR5] Bird, S. (2006). NLTK: The Natural Language Toolkit. In Proceedings of the COLING/ACL 2006 Interactive Presentation Sessions, pp. 69–72. Association for Computational Linguistics. 10.3115/1225403.1225421. https://aclanthology.org/P06-4018.

[CR6] Bombieri, M., Rospocher, M., Dall’Alba, D., & Fiorini, P. (2021). Automatic detection of procedural knowledge in robotic-assisted surgical texts. International Journal of Computer Assisted Radiology and Surgery **16**. 10.1007/s11548-021-02370-9. 10.1007/s11548-021-02370-9 PMC8295094 33886045

[CR7] Bombieri, M., Rospocher, M., Ponzetto, S.P., & Fiorini, P. (2022). The robotic surgery procedural framebank. In Proceedings of the Thirteenth International Conference on Language Resources and Evaluation (LREC 2022). European Language Resources Association (ELRA).

[CR8] Bombieri, M., Rospocher, M., Ponzetto, S. P., & Fiorini, P. (2023). Machine understanding surgical actions from intervention procedure textbooks. *Computers in Biology and Medicine,**152*, 106415. 10.1016/j.compbiomed.2022.106415 36527782 10.1016/j.compbiomed.2022.106415

[CR9] Campos, R., Mangaravite, V., Pasquali, A., Jorge, A., Nunes, C., & Jatowt, A. (2020). Yake! keyword extraction from single documents using multiple local features. *Information Sciences,**509*, 257–289. 10.1016/j.ins.2019.09.013

[CR10] Carreras, X., & Màrquez, L. (2005). Introduction to the conll-2005 shared task: semantic role labeling. In Dagan, I., Gildea, D. (eds.) Proceedings of the ninth conference on computational natural language learning, CoNLL 2005, June 29–30, 2005, pp. 152–164. ACL. https://aclanthology.org/W05-0620/.

[CR11] Chen, X., Xie, H., Wang, F. L., Liu, Z., Xu, J., & Hao, T. (2018). A bibliometric analysis of natural language processing in medical research. *BMC Medical Informatics Decision Making,**18*(1), 14–11414. 10.1186/s12911-018-0594-x 29589569 10.1186/s12911-018-0594-x PMC5872501

[CR12] Chou, W.-C., Tsai, R.T.-H., Su, Y.-S., Ku, W., Sung, T.-Y., & Hsu, W.-L. (2006). A semi-automatic method for annotating a biomedical Proposition Bank. In Proceedings of the Workshop on Frontiers in Linguistically Annotated Corpora 2006, pp. 5–12. Association for Computational Linguistics. https://aclanthology.org/W06-0602.

[CR13] Hasan, K.S., & Ng, V. (2014). Automatic Keyphrase extraction: A survey of the state of the art. In Proceedings of the 52nd Annual Meeting of the Association for Computational Linguistics (Volume 1: Long Papers), pp. 1262–1273. Association for Computational Linguistics. 10.3115/v1/P14-1119. https://aclanthology.org/P14-1119

[CR14] Houssein, E. H., Mohamed, R. E., & Ali, A. A. (2021). Machine learning techniques for biomedical natural language processing: A comprehensive review. *IEEE Access,**9*, 140628–140653. 10.1109/ACCESS.2021.3119621

[CR15] Hovy, E., Marcus, M., Palmer, M., Ramshaw, L., & Weischedel, R. (2006). OntoNotes: The 90% solution. In Proceedings of the human language technology conference of the NAACL, companion volume: short papers, pp. 57–60. Association for Computational Linguistics. https://aclanthology.org/N06-2015.

[CR16] Jiang, Y., Zaporojets, K., Deleu, J., Demeester, T., & Develder, C. (2020). Recipe instruction semantics corpus (risec): Resolving semantic structure and zero anaphora in recipes. In Wong, K., Knight, K., Wu, H. (eds.) Proceedings of the 1st Conference of the Asia-Pacific Chapter of the Association for Computational Linguistics and the 10th International Joint Conference on Natural Language Processing, AACL/IJCNLP 2020, December 4–7, 2020, pp. 821–826. Association for Computational Linguistics. https://aclanthology.org/2020.aacl-main.82/.

[CR17] Jindal, I., Rademaker, A., Ulewicz, M., Linh, H., Nguyen, H., Tran, K.-N., Zhu, H., & Li, Y. (2022). Universal proposition bank 2.0. In: Proceedings of the language resources and evaluation conference, pp. 1700–1711. European Language Resources Association https://aclanthology.org/2022.lrec-1.181

[CR18] Kara, N., Aslan, D.B., Marşan, B., Bakay, Ö., Ak, K., & Yıldız, O.T. (2020). Tropbank: Turkish propbank v2.0. In Proceedings of The 12th Language Resources and Evaluation Conference, pp. 2763–2772. European Language Resources Association. https://www.aclweb.org/anthology/2020.lrec-1.336.

[CR19] Klie, J.-C., Bugert, M., Boullosa, B., de Castilho, R.E., & Gurevych, I. (2018). The inception platform: Machine-assisted and knowledge-oriented interactive annotation. In Proceedings of the 27th International Conference on Computational Linguistics: System Demonstrations, pp. 5–9. Association for Computational Linguistics. Event Title: The 27th International Conference on Computational Linguistics (COLING 2018). http://tubiblio.ulb.tu-darmstadt.de/106270/.

[CR20] Li, T., Jawale, P.A., Palmer, M., Srikumar, V.: Structured tuning for semantic role labeling. In Jurafsky, D., Chai, J., Schluter, N., & Tetreault, J.R. (eds.) (2020). Proceedings of the 58th Annual Meeting of the Association for Computational Linguistics, ACL 2020, Online, July 5–10, 2020, pp. 8402–8412. Association for Computational Linguistics, United States. 10.18653/v1/2020.acl-main.744.

[CR21] Locke, S., Bashall, A., Al-Adely, S., Moore, J., Wilson, A., & Kitchen, G. B. (2021). Natural language processing in medicine: A review. *Trends in Anaesthesia and Critical Care,**38*, 4–9. 10.1016/j.tacc.2021.02.007

[CR22] Majewska, O., Collins, C., Baker, S., Björne, J., Brown, S. W., Korhonen, A., & Palmer, M. (2021). Bioverbnet: A large semantic-syntactic classification of verbs in biomedicine. *Journal of Biomedical Semantics,**12*(1), 12. 10.1186/s13326-021-00247-z 34266499 10.1186/s13326-021-00247-z PMC8280585

[CR23] McHugh, M. L. (2012). Interrater reliability: The kappa statistic. *Biochemia Medica,**22*, 276–282. 23092060 PMC3900052

[CR24] Mirzaei, A., & Moloodi, A. (2016). Persian proposition bank. In Chair), In N.C.C., Choukri, K., Declerck, T., Goggi, S., Grobelnik, M., Maegaard, B., Mariani, J., Mazo, H., Moreno, A., Odijk, J., Piperidis, S. (eds.) Proceedings of the Tenth International Conference on Language Resources and Evaluation (LREC 2016). European Language Resources Association (ELRA)

[CR25] Moeller, S.R., Wagner, I., Palmer, M., Conger, K., & Myers, S. (2020). The russian propbank. In Calzolari, N., Béchet, F., Blache, P., Choukri, K., Cieri, C., Declerck, T., Goggi, S., Isahara, H., Maegaard, B., Mariani, J., Mazo, H., Moreno, A., Odijk, J., Piperidis, S. (eds.) Proceedings of The 12th language resources and evaluation conference, LREC 2020, May 11–16, 2020, pp. 5995–6002. European Language Resources Association. https://aclanthology.org/2020.lrec-1.734/.

[CR26] Mysore, S., Jensen, Z., Kim, E., Huang, K., Chang, H.-S., Strubell, E., Flanigan, J., McCallum, A., & Olivetti, E. (2019). The materials science procedural text corpus: Annotating materials synthesis procedures with shallow semantic structures. In: Proceedings of the 13th Linguistic Annotation Workshop, pp. 56–64. Association for Computational Linguistics. 10.18653/v1/W19-4007. https://aclanthology.org/W19-4007.

[CR27] Palmer, M., Kingsbury, P. R., & Gildea, D. (2005). The proposition bank: An annotated corpus of semantic roles. *Computational Linguistics,**31*(1), 71–106. 10.1162/0891201053630264

[CR28] Peng, Y., Zhang, Z., Wang, X., Yang, L., & Lu, L. (2020). Chapter 5-text mining and deep learning for disease classification. The Elsevier and MICCAI society book seriesIn S. K. Zhou, D. Rueckert, & G. Fichtinger (Eds.), *Handbook of medical image computing and computer assisted intervention. *Academic Press.

[CR29] Schuler, K.K. (2006). Verbnet: A broad-coverage, comprehensive verb lexicon. PhD thesis, University of Pennsylvania.

[CR30] Shi, P., & Lin, J. (2019). Simple BERT models for relation extraction and semantic role labeling. CoRR arXiv:1904.05255.

[CR31] Surdeanu, M., Johansson, R., Meyers, A., Màrquez, L., & Nivre, J. (2008). The conll 2008 shared task on joint parsing of syntactic and semantic dependencies. In Clark, A., Toutanova, K. (eds.) Proceedings of the twelfth conference on computational natural language learning, CoNLL 2008, August 16–17, 2008, pp. 159–177. ACL https://aclanthology.org/W08-2121/.

[CR32] Taylor, R. H., Menciassi, A., Fichtinger, G., Fiorini, P., & Dario, P. (2016). Medical robotics and computer-integrated surgery. In B. Siciliano & O. Khatib (Eds.), *Springer handbook of robotics* (pp. 1657–1684). Springer. 10.1007/978-3-319-32552-1_63

[CR33] Varvara, R. (2017). Verbs as nouns: empirical investigations on event-denoting nominalizations. PhD thesis, University of Trento.

[CR34] Wang, Y. (2015). Semantic information extraction for software requirements using semantic role labeling. In 2015 IEEE International Conference on Progress in Informatics and Computing (PIC), pp. 332–337. 10.1109/PIC.2015.7489864.

[CR35] Wang, Y., Pakhomov, S., & Melton, G. (2013). Predicate argument structure frames for modeling information in operative notes. In MEDINFO 2013 - Proceedings of the 14th World Congress on Medical and Health Informatics. Studies in Health Technology and Informatics, pp. 783–787. IOS Press. 10.3233/978-1-61499-289-9-783. 14th World Congress on Medical and Health Informatics, MEDINFO 2013 ; Conference date: 20-08-2013 Through 23-08-2013. PMC4662251 23920664

[CR36] Weischedel, R.M., Hovy, E.H., Marcus, M.P., & Palmer, M. (2017). Ontonotes : A large training corpus for enhanced processing. In Handbook of Natural Language Processing and Machine Translation: DARPA Global Autonomous Language Exploitation. Springer

[CR37] Zhang, J., Nie, Y., Chang, J., & Zhang, J. (2021). Surgical instruction generation with transformers. In de Bruijne, M., Cattin, P.C., Cotin, S., Padoy, N., Speidel, S., Zheng, Y., Essert, C. (eds.) Medical image computing and computer assisted intervention-MICCAI 2021-24th International Conference, Strasbourg, France, September 27–October 1, 2021, Proceedings, Part IV. Lecture Notes in Computer Science, vol. 12904, pp. 290–299. Springer. 10.1007/978-3-030-87202-1_28.

[CR38] Zhang, Z., Webster, P., Uren, V., Varga, A., & Ciravegna, F. (2012). Automatically extracting procedural knowledge from instructional texts using natural language processing. In Proceedings of the Eighth International Conference on Language Resources and Evaluation (LREC’12), pp. 520–527. European Language Resources Association (ELRA). http://www.lrec-conf.org/proceedings/lrec2012/pdf/244_Paper.pdf

